# Prior subacromial decompression is a significant risk factor for development of acromial stress fracture after reverse total shoulder arthroplasty

**DOI:** 10.1016/j.jseint.2025.05.014

**Published:** 2025-06-02

**Authors:** Patrick E. Saunders, Clayton Hui, Abhay Mathur, Edward J. Quilligan, Hafiz F. Kassam

**Affiliations:** a Department of Orthopedic Education and Research, Hoag Orthopedics, Irvine, CA, USA; b College of Medicine, University of Arizona, Phoenix, AZ, USA; c Department of Orthopedic Surgery, Hoag Orthopedic Institute, Newport Beach, CA, USA

**Keywords:** Reverse shoulder arthroplasty, Acromial stress fracture, Risk factors, Complication, Subacromial decompression, Acromioplasty

## Abstract

**Background:**

As the use of reverse total shoulder arthroplasty (rTSA) has steadily increased since Food and Drug Administration approval in 2003, there have been a number of unique complications recognized, including postoperative acromial stress fractures (ASFs). The incidence of ASF after rTSA is rare; however, the impact on clinical outcomes and patient satisfaction can be devastating. Despite a growing body of literature exploring risk factors for ASF following rTSA, there has been minimal investigation into prior subacromial decompression/acromioplasty (SAD) as a risk factor. The purpose of this study was to review a large patient database to determine if prior SAD increases the risk for ASF after rTSA.

**Methods:**

The PearlDiver database was used to perform a retrospective cohort study of patients undergoing primary rTSA between 2010 and 2022. International Classification of Diseases and Current Procedural Terminology codes were used to identify patients undergoing rTSA and determine the incidence of ASF. Demographic characteristics and independent risk factors, including prior SAD, were compared between patients with and without ASFs.

**Results:**

A total of 106,599 patients undergoing primary rTSA were identified. The overall incidence of ASF was 0.90%. Prior SAD was identified as a significant independent risk factor with logistic regression analysis showing that prior SAD conferred a 26% higher risk of sustaining a postoperative ASF (odds ratio, 1.26 [95% confidence interval, 1.03-1.54]; *P* < .01). Additional independent risk factors for ASF following rTSA included increased Charlson Comorbidity Index, history of a rotator cuff tear, osteoporosis and inflammatory arthropathy.

**Conclusion:**

This study represents one of the largest cohorts of ASFs to date and is the first database study specifically investigating prior SAD as an independent risk factor for ASF after rTSA. Our results support that prior SAD is indeed an independent risk factor for ASF after rTSA. Further high quality, multicenter studies investigating prior SAD as a risk factor for ASF following rTSA are needed as our results supplement a sparse compendium of literature with mixed results pertaining to the hazard that SAD poses for development of this rare complication.

Since its approval by the Food and Drug Administration in 2003, the incidence of reverse total shoulder arthroplasty (rTSA) has steadily increased. Between 2011 and 2017 the number of primary rTSAs performed in the United Sates surged by nearly 200%, and projection models indicate this trend will continue going forward.^23^ As the use of rTSA has increased, there have been a number of unique complications recognized, including postoperative acromial stress fractures (ASFs).^6^ The incidence of an ASF after rTSA is somewhat rare, with studies demonstrating an incidence between 2 and 5%.^4^^,^^11^, ^12^, ^13^, ^14^^,^^16^ However, their impact on clinical outcomes and patient satisfaction can be devastating. Recent literature has demonstrated that patients experience significantly worse clinical, and patient-reported outcomes after rTSA if they sustain an ASF.^3^^,^^5^^,^^11^

As rTSA use continues to increase, there is a growing body of literature related to potential risk factors for postoperative ASF. Certain patient specific demographic risk factors such age, biologic sex, bone quality and underlying medical comorbidities have been shown to influence the risk of sustaining an ASF.^3^^,^^5^^,^^12^^,^^14^, ^15^, ^16^^,^^21^^,^^22^^,^^25^^,^^26^ Specifically, osteoporosis,^12^^,^^13^^,^^16^^,^^21^^,^^22^ inflammatory arthropathies,^12^^,^^13^^,^^16^^,^^21^ and rotator cuff tear/cuff tear arthropathy^13^^,^^16^^,^^22^ have all been shown to increase the risk of postoperative ASF. Furthermore, variations in prosthesis design have also been investigated and shown to impact the risk of sustaining this troublesome complication,^7^^,^^9^^,^^17^^,^^18^ such as increased risk of fracture with the use of an onlay humeral stem.^7^ There have even been recent studies showing that differences in underlying acromial morphology may influence a patient's risk of sustaining an ASF after rTSA.^8^^,^^20^

Despite a growing body of literature exploring various risk factors for ASF following rTSA, there is a paucity of literature investigating the impact of prior subacromial decompression/acromioplasty (SAD) as a potential risk factor. The results of the limited prior research on this topic are contradictory as to whether or not prior SAD increases the risk for ASF following rTSA, with some results suggesting increased risk for fracture in the setting of prior SAD/acromioplasty,^14^ whereas others demonstrate no increase in risk.^2^ These studies are limited by small patient cohorts owing to the rarity of this complication. As such, the purpose of this study was to utilize a large insurance claims database to determine if prior SAD increases the risk of sustaining an ASF following rTSA. We hypothesize that prior SAD does significantly increase the risk for postoperative ASF following rTSA.

## Methods

### Study design

The PearlDiver Mariner dataset was used to perform a retrospective cohort study. The Mariner dataset is an expansive national dataset of pooled insurance claims in the United States which contains deidentified data related to patient demographic characteristics, medical diagnoses, and inpatient/outpatient procedures. This particular dataset encompasses 170 million patients. The patient cohort in this study was isolated by querying the dataset from 2010 to 2022.

### Patient selection

International Classification of Diseases, Ninth Revision (ICD-9) and International Classification of Diseases, 10th Revision (ICD-10) codes were used to identify patients who had undergone primary rTSA (Supplementary Table S1). Patients with a diagnosis of acromial fracture or scapular fracture prior to undergoing rTSA or patients undergoing revision shoulder arthroplasty were excluded. The resultant cohort was subsequently divided into those with and without an ASF using ICD-9 and ICD-10 codes (Supplementary Table S2). As laterality is not detailed by Current Procedural Terminology (CPT) or ICD-9 codes, we had to assume that SAD, rTSA, and ASF all occurred on the same extremity for each patient included.

### Identification of comorbidities

Demographic characteristics including age and biologic sex were collected for all patients (Table I). The Charlson Comorbidity Index (CCI) was calculated for all patients to serve as a surrogate for overall health and extent of comorbid conditions (Table I). The CCI is a comorbidity index that predicts 10-year survival in patients with multiple comorbidities. It consists of 19 comorbidities and was collected for each patient through the database query. Relevant associated diagnoses for each patient including osteopenia, osteoporosis, osteoarthritis, inflammatory arthropathies, and rotator cuff tears were also collected using ICD-9 and ICD-10 codes (Supplementary Table S3). CPT codes (Supplementary Table S4) were used to determine if patients had undergone a SAD prior to undergoing rTSA.

**Table I:** Demographic characteristics of patient with and without acromial stress fracture (ASF) following reverse total shoulder arthroplasty.

	No ASF, n = 105,642	ASF, n = 957	*P* value
Age, mean (SD), yr	70.8 (10.4)	71.4 (10.7)	.08
Biologic sex, female	66,231 (62.7)	655 (68.4)	**<.01**
Charlson Comorbidity Index	2.44 (2.35)	2.77 (2.43)	**<.01**

*SD*, standard deviation.

Bold means the *P*-value has reached statistical significance.

### Statistical analysis

All statistical analyses were performed using Stata software (version 17; StataCorp, College Station, TX, USA) or R statistical software (version 4.1; R Foundation for Statistical Computing, Vienna, Austria) incorporated within PearlDiver. Descriptive statistics including means and proportions were conveyed, and bivariate analyses were performed using the Pearson's chi-squared tests or Fisher's exact test for categorical variables and the *t*-test for continuous variables. Significance was set at *P* = .05 for demographic characteristics, comorbidity, and relevant associated diagnoses comparisons between patients with and without ASFs. Multivariate logistic regression was implemented to identify independent predictors of ASF. All demographic characteristics, comorbidities, and relevant associated diagnoses were included in the multivariate logistic regression. The results of the multivariate logistic regression were reported as adjusted odds ratios (ORs) with 95% confidence intervals (CIs). The significance level for the multivariate logistic regression model was set at *P* = .05.

## Results

### Patient demographic characteristics and comorbidities

A total of 106,599 primary rTSAs were identified between 2010 and 2022. Of these, 957 patients sustained an ASF (0.90%). The demographic characteristics of patients with and without acromial factures are shown in Table I. The average age of those sustaining an ASF was 71.4 years compared to 70.8 years in those who did not. This difference was not significant (*P* = .28). The proportion of biologic females in the cohort sustaining ASFs was significantly higher than the cohort of patients who did not sustain an ASF (68.4% vs 62.7%; *P* < .01). The average CCI was significantly higher in patients who sustained an ASF compared to those who did not (2.77 vs 2.44; *P* < .01). The prevalence of comorbid diagnoses in each cohort are shown in Table II.

**Table II:** Associated diagnoses/comorbidities of patients with and without acromial stress fracture (ASF) following reverse total shoulder arthroplasty.

	No ASF, n = 105,642	ASF, n = 957	*P* value
Subacromial decompression	8,636 (8.2)	119 (12.4)	**<.01**
Rotator cuff tear	42,698 (40.4)	523 (54.6)	**<.01**
Osteoarthritis	75,776 (71.7)	750 (78.4)	**<.01**
Osteopenia	16,840 (15.9)	174 (18.2)	.06
Osteoporosis	21,445 (20.3)	322 (33.6)	**<.01**
Inflammatory arthropathy	18,082 (17.1)	249 (26)	**<.01**

Bold means the *P*-value has reached statistical significance.

### Independent predictors of ASF

Multivariate logistic regression revealed several independent predictors of ASF (Table III). Prior SAD (OR, 1.26 [95% CI, 1.03-1.54]; *P* < .01), increased CCI (OR, 1.03 [95% CI, 1.01-1.06]; *P* < .01), history of rotator cuff tear (OR, 1.57 [95% CI, 1.36-1.80]; *P* < .01), osteoporosis (OR, 1.86 [95% CI, 1.60-2.15]; *P* < .01), and inflammatory arthropathy (OR, 1.38 [95% CI, 1.18-1.61]; *P*, .001) all served as significant independent risk factors for ASF based on our analysis. Increased age, female sex, osteoarthritis, and osteopenia did not confer an increased risk.

**Table III:** Multivariate logistic regression results of independent risk factors for development of acromial stress fracture following reverse total shoulder arthroplasty.

Variable	Odds ratios	*P* value
Age	0.99 [0.98-1.01]	.67
CCI	1.03 [1.01-1.06]	**.01**
Biologic sex, female	1.13 [0.98-1.31]	.10
SAD	1.26 [1.03-1.54]	**.02**
Rotator cuff tear	1.57 [1.36-1.80]	**<.01**
Osteoarthritis	1.07 [0.91-1.27]	.40
Osteopenia	0.95 [0.80-1.13]	.59
Osteoporosis	1.86 [1.60-2.15]	**<.01**
Inflammatory arthropathy	1.38 [1.18-1.61]	**<.01**

*CCI*, Charlson Comorbidity Index; *SAD*, subacromial decompression/acromioplasty.

Bold means the *P*-value has reached statistical significance.

## Discussion

This retrospective study using deidentified data obtained from a national database of pooled insurance claims identified 106,599 patients who underwent primary rTSA between 2010 and 2022. Of these, 957 patients were identified as having sustained a postoperative ASF. The size of the overall patient cohort, as well as the magnitude of the cohort of patients who sustained an ASF, are among the largest in the literature to date. The overall incidence of ASF following primary rTSA was 0.90%. Most notably, history of a prior SAD was an independent risk factor for developing an ASF. In addition, multiple underlying conditions and diagnoses were found to increase the risk for development of an ASF including increased CCI, history of rotator cuff tear, osteoporosis, and inflammatory arthropathy. Of note, osteoporosis was the comorbid condition that posed the highest increased risk for development of an ASF (OR, 1.86 [95% CI, 1.60-2.15]; *P* < .01) with multivariate logistic regression.

The incidence of ASF in our study is marginally lower than the range (1.4%-5%) established in prior studies.^4^^,^^11^, ^12^, ^13^, ^14^^,^^16^^,^^21^ Su et al recently performed a database study investigating ASF after primary rTSA and similarly found that their results established an incidence (1.4%) of ASF that was somewhat lower than prior studies had reported.^21^ They note that their incidence may better represent the true prevalence of this infrequent complication given the much larger population assembled through the use of a database compared to prior cohort studies. They also offer that the discrepancy in reported incidence could be due to miscoding or noncoding of ASFs by providers. We feel that their line of reasoning is valid and sound and likewise applies to the comparatively lower incidence of ASF that our study found given the similar methodologies of our investigations. That being said, we acknowledge that the opposite could also be argued – lack of coding or miscoding by providers could have ultimately led to an underestimation of the incidence of ASFs by our methods.

While multivariate logistic regression demonstrated that multiple factors were significantly associated with an increased risk for the development of ASF after rTSA, we were particularly interested to discover that a history of prior SAD resulted in increased risk of ASF. In the cohort that sustained an ASF, 12.4% of the patients had a history of prior SAD, whereas in the cohort without an ASF, only 8.2% of the patients had a history of prior SAD. This difference in prevalence of prior SAD between these cohorts was statistically significant. Furthermore, logistic regression showed that as an independent risk factor prior SAD conferred roughly 26% higher risk of sustaining a postoperative ASF (OR, 1.26 [95% CI, 1.03-1.54]; *P* < .01). To our knowledge, this is the first database study to investigate SAD as a risk factor for ASF following rTSA, and this investigation contains one of the largest cohorts of ASFs in the literature to date.

There are limited prior investigations looking into SAD as a risk factor for ASF, and the results are mixed. Blaber et al conducted a retrospective matched-cohort study comparing outcomes of rTSA in patients with prior arthroscopic acromioplasty vs a control group of patients with no history of acromioplasty, and they found that there was no difference in postoperative acromial fracture rate between cases and controls.^2^ Marigi et al performed a retrospective review to assess the prevalence and risk factors for acromial facture after primary rTSA.^14^ Their results showed there was a trend toward increased risk of fracture for patients with prior acromioplasty, with 36% of patients in the acromial fracture groups having history of prior acromioplasty vs only 3% in the cohort that did not sustain an acromial fracture (*P* = .07). Not only did our study results reach statistical significance, but the size of our cohort is far larger than those of the 2 aforementioned investigations.

There have been a number of recent studies looking into the role that acromial morphology and acromial compromise may play in developing ASF after rTSA. Archambault et al performed a case control study to evaluate the role of anatomic scapular morphology in acromion and scapular spine fracture risk after rTSA.^1^ They analyzed three-dimensional computed tomography scans of 149 patients who underwent rTSA over a 10-year period and discovered that 51 of them sustained acromial fracture after rTSA. The authors found that patients with thicker acromions and thinner medial scapular spines had an increased fracture risk. Sabesan et al performed a parametric study that coupled three-dimensional shoulder kinematic simulation and three-dimensional finite element analysis to examine the effects of acromion size on the fatigue life and fracture risk after rTSA.^20^ Their results indicated that there was an optimal acromion size (+2.5 mm compared to normal male) that led to the lowest fracture risk in rTSA, suggesting that increased acromion size may decrease the risk of ASF after rTSA. Kim et al performed a retrospective study to investigate if acquired acromial compromise, including thinning or fragmentation, affects functional outcome scores, range of motion, strength, and complication rate following rTSA.^10^ Their results showed that the risk of acromial insufficiency fracture was not different between patients with and without acromial compromise (13% [3 of 23] vs 6% [4 of 69], relative risk 2 [95% CI 1-9]; *P* = .36). Similarly, Werner et al conducted a retrospective case-control study of patients who underwent rTSA to determine the effect of preoperative acromial compromise on outcomes following rTSA.^24^ Their study included 11 cases and 33 controls, and their results showed that at 2 years postoperatively there was no significant difference between the groups with regard to complications. In the context of these studies, our results possibly suggest that reduced acromial thickness increases the risk for ASF after rTSA; however, this cannot be definitively concluded. While not guaranteed, in some instances when a surgeon performs a subacromial decompression, this includes an acromioplasty, whereby the thickness of the acromion is diminished. As our results indicate greater risk of acromial fracture in the setting of prior SAD, there is the possibility that this may be due acromial compromise resulting from a variable degree of thinning of the acromion as part of the procedure. Overall, however, there is a lack of consensus in the literature regarding the role that SAD may play in escalating the risk for ASF after rTSA.

Our logistic regression identified a number of additional independent risk factors for ASF after rTSA that are in keeping with recent literature. Prior studies have consistently shown that osteoporosis,^12^^,^^13^^,^^16^^,^^21^^,^^22^ inflammatory arthropathies,^12^^,^^13^^,^^16^^,^^18^^,^^19^^,^^21^ and rotator cuff tear/cuff tear arthropathy^13^^,^^16^^,^^18^^,^^19^^,^^22^ are all associated with increased risk of ASF. Our results similarly demonstrate increased risk with each of these various diagnoses/conditions (Table III). Reduced bone mineral density and poor bone quality that result from osteoporosis and inflammatory arthropathies leave the acromion less capable of withstanding the altered forces it bears from increased deltoid tension which results from the mechanical design of rTSA protheses, leading to higher rates of postoperative fracture. In patients with a history of rotator cuff tear/cuff tear arthropathy, it is posited that chronic erosion of the inferior acromion from a high riding humeral head may lead to compromise of acromial integrity, which subsequently increases the risk for fracture.^4^ This postulation potentially underlies the increased risk of fracture that our results suggest with prior SAD as well – reduced acromial integrity due to possible thinning of the acromion during SAD potentially renders the acromion more susceptible to fracture after rTSA. However, not all subacromial decompression procedures include acromioplasty, and for those that do the extent of acromial thinning, is often minimal resection of an inferior spur; thus the strength of this conclusion must be considered in this context of uncertainty.

This study is not without limitations. First, while database studies provide the benefit of large patient populations, their use in performing research to answer clinical inquires is contingent on the accuracy of billing codes submitted by physicians. Miscoding, or failure to record codes at all, for any of the diagnoses/procedures we queried has the potential to lead to miscalculation and reduced validity of our results. Specifically, the low frequency of ASFs (0.90%) found in our study could be due to the fact that this complication occurs in the postoperative period and not all patients may have the appropriate diagnosis added to their chart, which ultimately may have led to an underestimation of incidence of ASFs. In general, the miscoding, or failure to code, certainly could have impacted the frequency of ASFs and SAD procedures captured in our results and ultimately the association between them. Next, laterality is not detailed by CPT or ICD-9 codes. We assumed that SAD, rTSA, and ASF all occurred on the same extremity for each patient included. This may not be accurate, and in some patients these codes may not have applied to the same extremity, in which case our final results may be skewed due to this assumption. Furthermore, we did not delineate the diagnosis that was tied to the indication for rTSA. As there are varying levels of risk for ASF reported for distinctive diagnoses leading to rTSA, this lack of delineation may have confounded our results. Finally, an additional potential confounding factor that we were unable to account for was the type of prostheses used. It has been reliably shown that certain aspects of prosthesis design impact the risk of ASF after rTSA; however, this information was not available to us through the database that was queried.

## Conclusion

ASFs following rTSA are rare, but troublesome complications that have been shown to have a substantially negative impact on patient outcomes. This study represents one of the largest cohorts of ASFs to date, and it is the first database study to specifically investigate prior SAD as an independent risk factor for ASF after rTSA. Our results support that prior SAD is an independent risk factor for the development of an ASF after rTSA. Further high quality, multicenter studies investigating prior SAD as an independent risk factor for ASF following rTSA are needed as our results supplement a sparse compendium of literature with mixed results pertaining to the hazard that SAD poses for development of this rare complication.

## Disclaimers

Funding: No funding was disclosed by the authors.

Conflicts of interest: The authors, their immediate families, and any research foundation with which they are affiliated have not received any financial payments or other benefits from any commercial entity related to the subject of this article.
