# Supplementary material for: Prior subacromial decompression is a significant risk factor for development of acromial stress fracture after reverse total shoulder arthroplasty
Source: JSES Int. 2025 Jun 2;9(5):1678–82. doi: 10.1016/j.jseint.2025.05.014 (PMC12490571; doi:10.1016/j.jseint.2025.05.014)
Supplement: Supplementary Table S1 [file mmc1.docx]

**Supplementary Table 1: ICD-9 and ICD-10 codes utilized to identify reverse total shoulder arthroplasty**

**ICD-9 Codes**

- **81.88**: Reverse total shoulder replacement

**ICD-10 Codes**

- **0RRJ00Z**: Replacement of Right Shoulder Joint with Reverse Ball and Socket Synthetic Substitute, Open Approach
- **0RRK00Z:** Replacement of Left Shoulder Joint with Reverse Ball and Socket Synthetic Substitute, Open Approach
