# Supplementary material for: Prior subacromial decompression is a significant risk factor for development of acromial stress fracture after reverse total shoulder arthroplasty
Source: JSES Int. 2025 Jun 2;9(5):1678–82. doi: 10.1016/j.jseint.2025.05.014 (PMC12490571; doi:10.1016/j.jseint.2025.05.014)
Supplement: Supplementary Table S2 [file mmc2.docx]

**Supplementary Table 2: ICD-9 and ICD-10 codes utilized to identify acromial stress fractures**

**ICD-9 Codes**

- **811.01**: Closed fracture of acromial process of scapula
- **811.11**: Open fracture of acromial process of scapula

**ICD-10 Codes**

- **S42.121A** - Displaced fracture of acromial process, right shoulder, initial encounter for closed fracture
- **S42.121B** - Displaced fracture of acromial process, right shoulder, initial encounter for open fracture
- **S42.121D** - Displaced fracture of acromial process, right shoulder, subsequent encounter for fracture with routine healing
- **S42.121G** - Displaced fracture of acromial process, right shoulder, subsequent encounter for fracture with delayed healing
- **S42.121K** - Displaced fracture of acromial process, right shoulder, subsequent encounter for fracture with nonunion
- **S42.121P** - Displaced fracture of acromial process, right shoulder, subsequent encounter for fracture with malunion
- **S42.121S** - Displaced fracture of acromial process, right shoulder, sequela
- **S42.122A** - Displaced fracture of acromial process, left shoulder, initial encounter for closed fracture
- **S42.122B** - Displaced fracture of acromial process, left shoulder, initial encounter for open fracture
- **S42.122D** - Displaced fracture of acromial process, left shoulder, subsequent encounter for fracture with routine healing
- **S42.122G** - Displaced fracture of acromial process, left shoulder, subsequent encounter for fracture with delayed healing
- **S42.122K** - Displaced fracture of acromial process, left shoulder, subsequent encounter for fracture with nonunion
- **S42.122P** - Displaced fracture of acromial process, left shoulder, subsequent encounter for fracture with malunion
- **S42.122S** - Displaced fracture of acromial process, left shoulder, sequela
- **S42.123A** - Displaced fracture of acromial process, unspecified shoulder, initial encounter for closed fracture
- **S42.123B -** Displaced fracture of acromial process, unspecified shoulder, initial encounter for open fracture
- **S42.123D** - Displaced fracture of acromial process, unspecified shoulder, subsequent encounter for fracture with routine healing
- **S42.123G** - Displaced fracture of acromial process, unspecified shoulder, subsequent encounter for fracture with delayed healing
- **S42.123K** - Displaced fracture of acromial process, unspecified shoulder, subsequent encounter for fracture with nonunion
- **S42.123P** - Displaced fracture of acromial process, unspecified shoulder, subsequent encounter for fracture with malunion
- **S42.123S** - Displaced fracture of acromial process, unspecified shoulder, sequela
- **S42.124A** - Nondisplaced fracture of acromial process, right shoulder, initial encounter for closed fracture
- **S42.124B** - Nondisplaced fracture of acromial process, right shoulder, initial encounter for open fracture
- **S42.124D** - Nondisplaced fracture of acromial process, right shoulder, subsequent encounter for fracture with routine healing
- **S42.124G** - Nondisplaced fracture of acromial process, right shoulder, subsequent encounter for fracture with delayed healing
- **S42.124K** - Nondisplaced fracture of acromial process, right shoulder, subsequent encounter for fracture with nonunion
- **S42.124P** - Nondisplaced fracture of acromial process, right shoulder, subsequent encounter for fracture with malunion
- **S42.124S** - Nondisplaced fracture of acromial process, right shoulder, sequela
- **S42.125A** - Nondisplaced fracture of acromial process, left shoulder, initial encounter for closed fracture
- **S42.125B** - Nondisplaced fracture of acromial process, left shoulder, initial encounter for open fracture
- **S42.125D** - Nondisplaced fracture of acromial process, left shoulder, subsequent encounter for fracture with routine healing
- **S42.125G** - Nondisplaced fracture of acromial process, left shoulder, subsequent encounter for fracture with delayed healing
- **S42.125K** - Nondisplaced fracture of acromial process, left shoulder, subsequent encounter for fracture with nonunion
- **S42.125P** - Nondisplaced fracture of acromial process, left shoulder, subsequent encounter for fracture with malunion
- **S42.125S** - Nondisplaced fracture of acromial process, left shoulder, sequela
- **S42.126A** - Nondisplaced fracture of acromial process, unspecified shoulder, initial encounter for closed fracture
- **S42.126B** - Nondisplaced fracture of acromial process, unspecified shoulder, initial encounter for open fracture
- **S42.126D** - Nondisplaced fracture of acromial process, unspecified shoulder, subsequent encounter for fracture with routine healing
- **S42.126G** - Nondisplaced fracture of acromial process, unspecified shoulder, subsequent encounter for fracture with delayed healing
- **S42.126K** - Nondisplaced fracture of acromial process, unspecified shoulder, subsequent encounter for fracture with nonunion
- **S42.126P** - Nondisplaced fracture of acromial process, unspecified shoulder, subsequent encounter for fracture with malunion
- **S42.126S** - Nondisplaced fracture of acromial process, unspecified shoulder, sequela
