# Supplementary material for: Prior subacromial decompression is a significant risk factor for development of acromial stress fracture after reverse total shoulder arthroplasty
Source: JSES Int. 2025 Jun 2;9(5):1678–82. doi: 10.1016/j.jseint.2025.05.014 (PMC12490571; doi:10.1016/j.jseint.2025.05.014)
Supplement: Supplementary Table S3 [file mmc3.docx]

**Supplementary Table 3: ICD-9 and ICD-10 codes utilized for identification of associated diagnoses/comorbidities**

**Osteopenia**

**ICD-9 Codes**

- **268.2**- Osteomalacia, unspecified
- **268.9** - Unspecified vitamin D deficiency

**ICD-10 Codes**

- **M83.1 -** Senile osteomalacia
- **M83.2 -** Adult osteomalacia due to malabsorption
- **M83.3 -** Adult osteomalacia due to malnutrition
- **M83.5 -** Other drug-induced osteomalacia in adults
- **M83.8 -** Other adult osteomalacia
- **M83.9 -** Adult osteomalacia, unspecified
- **M85.80 -** Other specified disorders of bone density and structure, unspecified site
- **M85.811 -** Other specified disorders of bone density and structure, right shoulder
- **M85.812 -** Other specified disorders of bone density and structure, left shoulder
- **M85.819 -** Other specified disorders of bone density and structure, unspecified shoulder
- **M85.821 -** Other specified disorders of bone density and structure, right upper arm
- **M85.822 -** Other specified disorders of bone density and structure, left upper arm
- **M85.829 -** Other specified disorders of bone density and structure, unspecified upper arm
- **M85.88 -** Other specified disorders of bone density and structure, other site
- **M85.89 -** Other specified disorders of bone density and structure, multiple sites
- **M85.9 -** Disorder of bone density and structure, unspecified

**Osteoporosis**

**ICD-9 Codes**

- **733.00** - Osteoporosis, unspecified
- **733.01** - Senile osteoporosis
- **733.02** - Idiopathic osteoporosis
- **733.03** - Disuse osteoporosis
- **733.09** - Other osteoporosis

**ICD-10 Codes**

- **M81.0 -** Age-related osteoporosis without current pathological fracture
- **M81.6 -** Localized osteoporosis
- **M81.8 -** Other osteoporosis without current pathological fracture
- **M80.00XA** - Age-related osteoporosis with current pathological fracture, unspecified site, initial encounter for fracture
- **M80.00XD -** Age-related osteoporosis with current pathological fracture, unspecified site, subsequent encounter for fracture with routine healing
- **M80.00XG -** Age-related osteoporosis with current pathological fracture, unspecified site, subsequent encounter for fracture with delayed healing
- **M80.00XK** - Age-related osteoporosis with current pathological fracture, unspecified site, subsequent encounter for fracture with nonunion
- **M80.00XP** - Age-related osteoporosis with current pathological fracture, unspecified site, subsequent encounter for fracture with malunion
- **M80.00XS** - Age-related osteoporosis with current pathological fracture, unspecified site, sequela
- **M80.011A -** Age-related osteoporosis with current pathological fracture, right shoulder, initial encounter for fracture
- **M80.011D -** Age-related osteoporosis with current pathological fracture, right shoulder, subsequent encounter for fracture with routine healing
- **M80.011G -** Age-related osteoporosis with current pathological fracture, right shoulder, subsequent encounter for fracture with delayed healing
- **M80.011K -** Age-related osteoporosis with current pathological fracture, right shoulder, subsequent encounter for fracture with nonunion
- **M80.011P -** Age-related osteoporosis with current pathological fracture, right shoulder, subsequent encounter for fracture with malunion
- **M80.011S -** Age-related osteoporosis with current pathological fracture, right shoulder, sequela
- **M80.012A** - Age-related osteoporosis with current pathological fracture, left shoulder, initial encounter for fracture
- **M80.012D -** Age-related osteoporosis with current pathological fracture, left shoulder, subsequent encounter for fracture with routine healing
- **M80.012G -** Age-related osteoporosis with current pathological fracture, left shoulder, subsequent encounter for fracture with delayed healing
- **M80.012K -** Age-related osteoporosis with current pathological fracture, left shoulder, subsequent encounter for fracture with nonunion
- **M80.012P -** Age-related osteoporosis with current pathological fracture, left shoulder, subsequent encounter for fracture with malunion
- **M80.012S** - Age-related osteoporosis with current pathological fracture, left shoulder, sequela
- **M80.019A** - Age-related osteoporosis with current pathological fracture, unspecified shoulder, initial encounter for fracture
- **M80.019D -** Age-related osteoporosis with current pathological fracture, unspecified shoulder, subsequent encounter for fracture with routine healing
- **M80.019G -** Age-related osteoporosis with current pathological fracture, unspecified shoulder, subsequent encounter for fracture with delayed healing
- **M80.019K –** Age-related osteoporosis with current pathological fracture, unspecified shoulder, subsequent encounter for fracture with nonunion
- **M80.019P -** Age-related osteoporosis with current pathological fracture, unspecified shoulder, subsequent encounter for fracture with malunion
- **M80.019S -** Age-related osteoporosis with current pathological fracture, unspecified shoulder, sequela

**Osteoarthritis**

**ICD-9 Codes**

- **715.11** - Osteoarthrosis, localized, primary, shoulder region
- **715.12** - Osteoarthrosis, localized, primary, upper arm
- **715.21** - Osteoarthrosis, localized, secondary, shoulder region
- **715.22** - Osteoarthrosis, localized, secondary, upper arm
- **715.31** - Osteoarthrosis, localized, not specified whether primary or secondary, shoulder region
- **715.32** - Osteoarthrosis, localized, not specified whether primary or secondary, upper arm
- **715.91** - Osteoarthrosis, unspecified whether generalized or localized, shoulder region
- **715.92** - Osteoarthrosis, unspecified whether generalized or localized, upper arm

**ICD-10 Codes**

- **M19.011 -** Primary osteoarthritis, right shoulder
- **M19.012 -** Primary osteoarthritis, left shoulder
- **M19.019 -** Primary osteoarthritis, unspecified shoulder
- **M19.211 -** Secondary osteoarthritis, right shoulder
- **M19.212 -** Secondary osteoarthritis, left shoulder
- **M19.219 -** Secondary osteoarthritis, unspecified shoulder

**Inflammatory/Reactive Arthropathies**

**ICD-9 Codes**

- **274.00** - Gouty arthropathy, unspecified
- **274.02** - Chronic gouty arthropathy without mention of tophus (tophi)
- **274.03** - Chronic gouty arthropathy with tophus (tophi)
- **696.0** - Psoriatic arthropathy
- **712.81** - Other specified crystal arthropathies, shoulder region
- **712.82** - Other specified crystal arthropathies, upper arm
- **712.91** - Unspecified crystal arthropathy, shoulder region
- **712.92** - Unspecified crystal arthropathy, upper arm
- **713.0** - Arthropathy associated with other endocrine and metabolic disorders
- **713.1** - Arthropathy associated with gastrointestinal conditions other than infections
- **713.2** - Arthropathy associated with hematological disorders
- **713.3** - Arthropathy associated with dermatological disorders
- **713.4** - Arthropathy associated with respiratory disorders
- **713.5** - Arthropathy associated with neurological disorders
- **713.7** - Other general diseases with articular involvement
- **713.8** - Arthropathy associated with other conditions classifiable elsewhere
- **714.0** - Rheumatoid arthritis
- **714.4** - Chronic postrheumatic arthropathy
- **714.89** - Other specified inflammatory polyarthropathies
- **714.9** - Unspecified inflammatory polyarthropathy
- **716.51** - Unspecified polyarthropathy or polyarthritis, shoulder region
- **716.52** - Unspecified polyarthropathy or polyarthritis, upper arm
- **716.61** - Unspecified monoarthritis, shoulder region
- **716.62** - Unspecified monoarthritis, upper arm
- **716.81** - Other specified arthropathy, shoulder region
- **716.82** - Other specified arthropathy, upper arm
- **716.91** - Arthropathy, unspecified, shoulder region
- **716.92** - Arthropathy, unspecified, upper arm

**ICD-10 Codes**

- **L40.50 -** Arthropathic psoriasis, unspecified
- **L40.59 -** Other psoriatic arthropathy
- **M1A.0110 -** Idiopathic chronic gout, right shoulder, without tophus (tophi)
- **M1A.0111 -** Idiopathic chronic gout, right shoulder, with tophus (tophi)
- **M1A.0120 -** Idiopathic chronic gout, left shoulder, without tophus (tophi)
- **M1A.0121 -** Idiopathic chronic gout, left shoulder, with tophus (tophi)
- **M1A.0190 -** Idiopathic chronic gout, unspecified shoulder, without tophus (tophi)
- **M1A.0191 -** Idiopathic chronic gout, unspecified shoulder, with tophus (tophi)
- **M02.311 -** Reiter's disease, right shoulder
- **M02.312 -** Reiter's disease, left shoulder
- **M02.319 -** Reiter's disease, unspecified shoulder
- **M02.811 -** Other reactive arthropathies, right shoulder
- **M02.812 -** Other reactive arthropathies, left shoulder
- **M02.819 -** Other reactive arthropathies, unspecified shoulder
- **M04.8 -** Other autoinflammatory syndromes
- **M04.9 -** Autoinflammatory syndrome, unspecified
- **M05.011 -** Felty's syndrome, right shoulder
- **M05.012 -** Felty's syndrome, left shoulder
- **M05.019 -** Felty's syndrome, unspecified shoulder
- **M05.111 -** Rheumatoid lung disease with rheumatoid arthritis of right shoulder
- **M05.112 -** Rheumatoid lung disease with rheumatoid arthritis of left shoulder
- **M05.119 -** Rheumatoid lung disease with rheumatoid arthritis of unspecified shoulder
- **M05.211 -** Rheumatoid vasculitis with rheumatoid arthritis of right shoulder
- **M05.212 -** Rheumatoid vasculitis with rheumatoid arthritis of left shoulder
- **M05.219 -** Rheumatoid vasculitis with rheumatoid arthritis of unspecified shoulder
- **M05.311 -** Rheumatoid heart disease with rheumatoid arthritis of right shoulder
- **M05.312 -** Rheumatoid heart disease with rheumatoid arthritis of left shoulder
- **M05.319 -** Rheumatoid heart disease with rheumatoid arthritis of unspecified shoulder
- **M05.411 -** Rheumatoid myopathy with rheumatoid arthritis of right shoulder
- **M05.412 -** Rheumatoid myopathy with rheumatoid arthritis of left shoulder
- **M05.419 -** Rheumatoid myopathy with rheumatoid arthritis of unspecified shoulder
- **M05.511 -** Rheumatoid polyneuropathy with rheumatoid arthritis of right shoulder
- **M05.512 -** Rheumatoid polyneuropathy with rheumatoid arthritis of left shoulder
- **M05.519 -** Rheumatoid polyneuropathy with rheumatoid arthritis of unspecified shoulder
- **M05.611 -** Rheumatoid arthritis of right shoulder with involvement of other organs and systems
- **M05.612 -** Rheumatoid arthritis of left shoulder with involvement of other organs and systems
- **M05.619 -** Rheumatoid arthritis of unspecified shoulder with involvement of other organs and systems
- **M05.711 -** Rheumatoid arthritis with rheumatoid factor of right shoulder without organ or systems involvement
- **M05.712 -** Rheumatoid arthritis with rheumatoid factor of left shoulder without organ or systems involvement
- **M05.719 -** Rheumatoid arthritis with rheumatoid factor of unspecified shoulder without organ or systems involvement
- **M05.811 -** Other rheumatoid arthritis with rheumatoid factor of right shoulder
- **M05.812 -** Other rheumatoid arthritis with rheumatoid factor of left shoulder
- **M05.819 -** Other rheumatoid arthritis with rheumatoid factor of unspecified shoulder
- **M06.011 -** Rheumatoid arthritis without rheumatoid factor, right shoulder
- **M06.012 -** Rheumatoid arthritis without rheumatoid factor, left shoulder
- **M06.019 -** Rheumatoid arthritis without rheumatoid factor, unspecified shoulder
- **M06.211 -** Rheumatoid bursitis, right shoulder
- **M06.212 -** Rheumatoid bursitis, left shoulder
- **M06.219 -** Rheumatoid bursitis, unspecified shoulder
- **M06.4 -** Inflammatory polyarthropathy
- **M06.80 -** Other specified rheumatoid arthritis, unspecified site
- **M06.811 -** Other specified rheumatoid arthritis, right shoulder
- **M06.812 -** Other specified rheumatoid arthritis, left shoulder
- **M06.819 -** Other specified rheumatoid arthritis, unspecified shoulder
- **M06.9 -** Rheumatoid arthritis, unspecified
- **M07.611 -** Enteropathic arthropathies, right shoulder
- **M07.612 -** Enteropathic arthropathies, left shoulder
- **M07.619 -** Enteropathic arthropathies, unspecified shoulder
- **M10.011 -** Idiopathic gout, right shoulder
- **M10.012 -** Idiopathic gout, left shoulder
- **M10.019 -** Idiopathic gout, unspecified shoulder
- **M10.411 -** Other secondary gout, right shoulder
- **M10.412 -** Other secondary gout, left shoulder
- **M10.419 -** Other secondary gout, unspecified shoulder
- **M11.811 -** Other specified crystal arthropathies, right shoulder
- **M11.812 -** Other specified crystal arthropathies, left shoulder
- **M11.819 -** Other specified crystal arthropathies, unspecified shoulder
- **M12.211 -** Villonodular synovitis (pigmented), right shoulder
- **M12.212 -** Villonodular synovitis (pigmented), left shoulder
- **M12.219 -** Villonodular synovitis (pigmented), unspecified shoulder
- **M12.811 -** Other specific arthropathies, not elsewhere classified, right shoulder
- **M12.812 -** Other specific arthropathies, not elsewhere classified, left shoulder
- **M12.819 -** Other specific arthropathies, not elsewhere classified, unspecified shoulder
- **M13.111 -** Monoarthritis, not elsewhere classified, right shoulder
- **M13.112 -** Monoarthritis, not elsewhere classified, left shoulder
- **M13.119 -** Monoarthritis, not elsewhere classified, unspecified shoulder
- **M13.811 -** Other specified arthritis, right shoulder
- **M13.812 -** Other specified arthritis, left shoulder
- **M13.819 -** Other specified arthritis, unspecified shoulder
- **M14.611 -** Charcôt's joint, right shoulder
- **M14.612 -** Charcôt's joint, left shoulder
- **M14.619 -** Charcôt's joint, unspecified shoulder
- **M14.811 -** Arthropathies in other specified diseases classified elsewhere, right shoulder
- **M14.812 -** Arthropathies in other specified diseases classified elsewhere, left shoulder
- **M14.819 -** Arthropathies in other specified diseases classified elsewhere, unspecified shoulder

**Rotator Cuff Tear**

**ICD-9 Codes**

- **726.13** - Partial tear of rotator cuff
- **727.61** - Complete rupture of rotator cuff

**ICD-10 Codes**

- **M75.100**- Unspecified rotator cuff tear or rupture of unspecified shoulder, not specified as traumatic
- **M75.101 -** Unspecified rotator cuff tear or rupture of right shoulder, not specified as traumatic
- **M75.102 -** Unspecified rotator cuff tear or rupture of left shoulder, not specified as traumatic
- **M75.110** - Incomplete rotator cuff tear or rupture of unspecified shoulder, not specified as traumatic
- **M75.111** - Incomplete rotator cuff tear or rupture of right shoulder, not specified as traumatic
- **M75.112** - Incomplete rotator cuff tear or rupture of left shoulder, not specified as traumatic
- **M75.120** - Complete rotator cuff tear or rupture of unspecified shoulder, not specified as traumatic
- **M75.121** - Complete rotator cuff tear or rupture of right shoulder, not specified as traumatic
- **M75.122** - Complete rotator cuff tear or rupture of left shoulder, not specified as traumatic
- **S46.001A** - Unspecified injury of muscle(s) and tendon(s) of the rotator cuff of right shoulder, initial encounter
- **S46.001D** - Unspecified injury of muscle(s) and tendon(s) of the rotator cuff of right shoulder, subsequent encounter
- **S46.001S** - Unspecified injury of muscle(s) and tendon(s) of the rotator cuff of right shoulder, sequela
- **S46.002A** - Unspecified injury of muscle(s) and tendon(s) of the rotator cuff of left shoulder, initial encounter
- **S46.002D** - Unspecified injury of muscle(s) and tendon(s) of the rotator cuff of left shoulder, subsequent encounter
- **S46.002S** - Unspecified injury of muscle(s) and tendon(s) of the rotator cuff of left shoulder, sequela
- **S46.009A** - Unspecified injury of muscle(s) and tendon(s) of the rotator cuff of unspecified shoulder, initial encounter
- **S46.009D** - Unspecified injury of muscle(s) and tendon(s) of the rotator cuff of unspecified shoulder, subsequent encounter
- **S46.009S** - Unspecified injury of muscle(s) and tendon(s) of the rotator cuff of unspecified shoulder, sequela
- **S46.021A** - Laceration of muscle(s) and tendon(s) of the rotator cuff of right shoulder, initial encounter
- **S46.021D** - Laceration of muscle(s) and tendon(s) of the rotator cuff of right shoulder, subsequent encounter
- **S46.021S** - Laceration of muscle(s) and tendon(s) of the rotator cuff of right shoulder, sequela
- **S46.022A** - Laceration of muscle(s) and tendon(s) of the rotator cuff of left shoulder, initial encounter
- **S46.022D** - Laceration of muscle(s) and tendon(s) of the rotator cuff of left shoulder, subsequent encounter
- **S46.022S** - Laceration of muscle(s) and tendon(s) of the rotator cuff of left shoulder, sequela
- **S46.029A** - Laceration of muscle(s) and tendon(s) of the rotator cuff of unspecified shoulder, initial encounter
- **S46.029D** - Laceration of muscle(s) and tendon(s) of the rotator cuff of unspecified shoulder, subsequent encounter
- **S46.029S** - Laceration of muscle(s) and tendon(s) of the rotator cuff of unspecified shoulder, sequela
- **S46.091A** - Other injury of muscle(s) and tendon(s) of the rotator cuff of right shoulder, initial encounter
- **S46.091D** - Other injury of muscle(s) and tendon(s) of the rotator cuff of right shoulder, subsequent encounter
- **S46.091S** - Other injury of muscle(s) and tendon(s) of the rotator cuff of right shoulder, sequela
- **S46.092A** - Other injury of muscle(s) and tendon(s) of the rotator cuff of left shoulder, initial encounter
- **S46.092D** - Other injury of muscle(s) and tendon(s) of the rotator cuff of left shoulder, subsequent encounter
- **S46.092S** - Other injury of muscle(s) and tendon(s) of the rotator cuff of left shoulder, sequela
- **S46.099A** - Other injury of muscle(s) and tendon(s) of the rotator cuff of unspecified shoulder, initial encounter
- **S46.099D** - Other injury of muscle(s) and tendon(s) of the rotator cuff of unspecified shoulder, subsequent encounter
- **S46.099S** - Other injury of muscle(s) and tendon(s) of the rotator cuff of unspecified shoulder, sequela
