# Supplementary material for: Prior subacromial decompression is a significant risk factor for development of acromial stress fracture after reverse total shoulder arthroplasty
Source: JSES Int. 2025 Jun 2;9(5):1678–82. doi: 10.1016/j.jseint.2025.05.014 (PMC12490571; doi:10.1016/j.jseint.2025.05.014)
Supplement: Supplementary Table S4 [file mmc4.docx]

**Supplementary Table 4: CPT codes utilized to identify prior subacromial decompression/acromioplasty**

**CPT Codes**

- **29826**: Arthroscopy, shoulder surgical; decompression of subacromial space with partial acromioplasty, with coracoacromial ligament release, when performed
- **23130**: Acromioplasty of acromionectomy, partial, with or without or without coracoacromial ligament release
- **23415**: Coracoacromial ligament release, with or without acromioplasty
